# Refractory Epstein-Barr Virus (EBV)-Related Post-transplant Lymphoproliferative Disease: Cure by Combined Brentuximab Vedotin and Allogeneic EBV-Specific T-Lymphocytes

**DOI:** 10.3389/fmed.2019.00295

**Published:** 2019-12-18

**Authors:** Thomas Mika, Katharina Strate, Swetlana Ladigan, Clemens Aigner, Uwe Schlegel, Iris Tischoff, Sabine Tischer-Zimmermann, Britta Eiz-Vesper, Britta Maecker-Kolhoff, Roland Schroers

**Affiliations:** ^1^Department of Medicine, Hematology and Oncology, Ruhr-University Bochum, Bochum, Germany; ^2^Department of Thoracic Surgery, Ruhrlandklinik, University Hospital Essen, Essen, Germany; ^3^Department of Neurology, Ruhr-University Bochum, Bochum, Germany; ^4^Department of Pathology, Ruhr-University Bochum, Bochum, Germany; ^5^Institute for Transfusion Medicine, Hannover Medical School, Hanover, Germany; ^6^Department of Pediatric Hematology and Oncology, Hannover Medical School, Hanover, Germany

**Keywords:** PTLD, alloHSCT, aggressive lymphoma, brentuximab vedotin (BV), third-party EBV-specific T-lymphocytes

## Abstract

Post-transplant lymphoproliferative disease (PTLD) represents a serious complication following allogeneic hematopoietic stem cell transplantation (alloHSCT). Previously, survival rates of PTLD have improved due to the introduction of rituximab. However, reports on curative management of refractory PTLD are scarce. Today, there is no consensus how to treat rituximab-refractory PTLD, especially in highly aggressive disease. Here, we describe successful management of refractory EBV-associated PTLD, specifically DLBCL, with combined brentuximab vedotin and third-party EBV-specific T-cells in a multidisciplinary treatment approach.

## Background

Post-transplant lymphoproliferative disease (PTLD) is a rare and serious complication after allogeneic hematopoietic stem cell transplantation (alloHSCT). PTLD is often linked to Epstein-Barr virus (EBV) infection or reactivation. It comprises a heterogeneous group ranging from polyclonal non-destructive to aggressive non-Hodgkin and Hodgkin-like disorders (1, 2). Previous studies have provided further insight into the nature and management of PTLD following alloHSCT (2, 3). During the past few years, survival rates of PTLD have improved, mainly due to the introduction of rituximab (1–3). However, reports on curative management of refractory PTLD are scarce.

Adoptive immunotherapy with EBV-specific T-lymphocytes directed against PTLD has shown promising results in different clinical trials (1, 2, 4). Furthermore, brentuximab vedotin (BV), a CD30-directed antibody-toxin conjugate, represents an attractive therapeutic approach due to CD30-expression in a subgroup of PTLD that is probably induced by EBV infection (1, 5). BV was clinically active in two studies on diffuse large B-cell lymphoma (DLBCL) with complete remission (CR) rates of 17 and 58%, respectively (6, 7). Currently, there is only limited reporting on the therapeutic application of BV in PTLD (8).

Here, we describe successful management of refractory EBV-associated PTLD, specifically DLBCL, with combined BV and third-party EBV-specific T-cells in a multidisciplinary treatment approach.

## Case Presentation – Description of Tests and Therapy

In November 2015, a 52-year old man presented with acute myeloid leukemia. Five years earlier, the patient had been treated with azathioprine for chronic inflammatory bowel disease (CIBD). Induction chemotherapy for AML comprised two cycles of cytarabine (700 mg/m^2^) and daunorubicine (180 mg/m^2^) and resulted in complete remission (CR). Following both cycles of the initial chemotherapy, the patient developed severe infections in neutropenia, requiring extensive antibiotic and antimycotic treatments.

The patient, who had AML in the intermediate-risk group according to European Leukemia Network (ELN) criteria, underwent alloHSCT following reduced-intensity conditioning with fludarabine (150 mg/m^2^) and treosulfan (30 g/m^2^) in the first CR. Graft-versus-Host disease (GvHD) prophylaxis consisted of ATG-F (60 mg/kg), methotrexate, and ciclosporine. Prior to alloHSCT, the patient was EBV-IgG and CMV-IgG seropositive; he received peripheral blood stem cells collected from an unrelated HLA-matched donor (10/10, MUD) who was EBV-IgG seronegative and CMV-IgG seropositive. At first, the chemotherapy and alloHSCT were well-tolerated. An episode of acute GvHD affecting the skin was successfully treated with short-term prednisolone.

Remarkably, the plasma EBV DNA, as determined by twice-weekly PCR monitoring assays, increased to 17.600 EBV copies/ml at day +88. However, the patient remained free of symptoms, and subsequent PCR measurements showed a drop to 11 EBV copies/ml within 3 days. Immunosuppression was discontinued at day +105. At this time point, the AML was in CR, and donor-chimerism was 100% in peripheral blood and bone marrow, respectively. No EBV copies were detected at this time point. At day +111, another short episode of clinically asymptomatic EBV DNAemia with 568.000 copies/ml, which decreased until less than 5 EBV copies/ml was detected.

**Figure 1:**
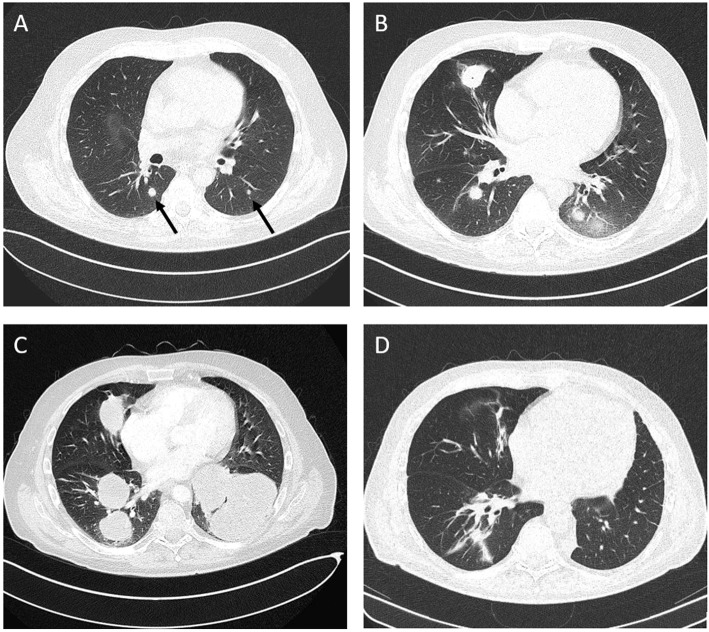
Computed tomography (CT), chest. **(A)** Disseminated atypical pulmonary infiltrates (day +145 after alloHSCT), **(B)** Chest CT following antimycotic therapy (day +170), **(C)** Disease progression after three courses of rituximab and two courses R-CHOP (day +228), **(D)** Complete remission (CR) after five courses of brentuximab vedotin and three courses of third-party EBV-specific T-cells.

**Figure 2:**
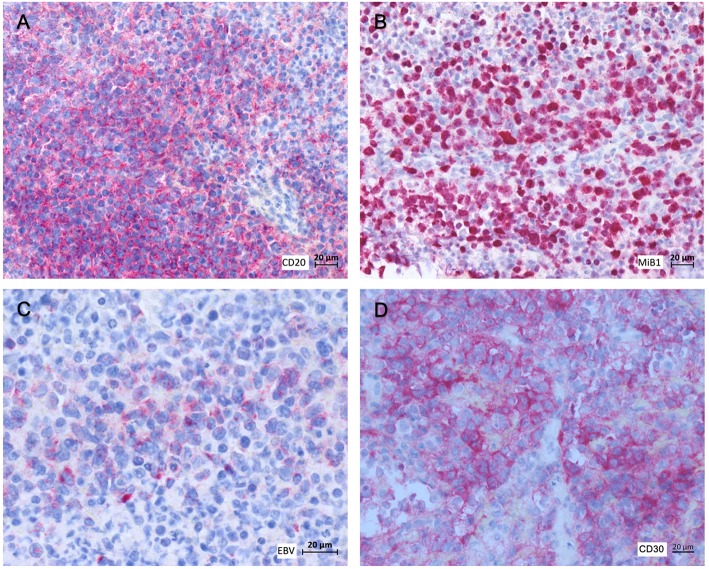
Histopathology and immunohistochemistry of pulmonary tumor biopsies. **(A)** αCD20, **(B)** MiB1, **(C)** αLMP1, **(D)** αCD30.

At day +145, the patient presented with fever and dry cough. A computed tomography (CT)-scan of the chest revealed disseminated pulmonary lesions (Figure 1A). Initially, fungal lung infection was deemed as the most likely cause, and, therefore, voriconazole was administered. However, no clinical improvement was observed, and CT-scan revealed rapid enlargement of the lesions (Figure 1B). Subsequent biopsy and histopathology gave the diagnosis of diffuse large B-cell lymphoma (DLBCL), displaying high cellular proliferation (MiB1 80%) and co-expression of EBV-derived proteins (LMP1), as well as CD20 and CD30 (Figure 2). Summarizing the results of histopathology, PET-CT staging (Figure 1A), and the history of EBV-reactivation (EBV-PCR negative at this time point) following alloHSCT, the diagnosis was monomorphic EBV-associated PTLD presenting as DLBCL in Ann Arbor stage IVB with multiple pulmonary lymphomas.

Despite immediate initiation of treatment with rituximab (375 mg/m^2^, weekly), progressive disease with increasing dyspnea was observed after three weekly cycles. Subsequently, two courses of R-CHOP14 were administered. However, along with deteriorating symptoms, another CT-scan documented further lymphoma progression (Figure 1C).

Recognizing the dismal prognosis of R-CHOP refractory DLBCL and the patient's impaired physical condition (ECOG 2-3), we decided against intensive second-line chemotherapy and started treatment with BV (1.8 mg/kg, monthly). At the same time, adoptive immunotherapy with third-party EBV-directed T-lymphocytes was prepared (alloCELL@mh-hannover.de). As previously described, the *in vitro-*selection of highly active EBV-specific T-lymphocytes is based on interferon-γ release following stimulation with EBNA-peptide pools (9, 10). Clinical-grade EBV-specific T-cells were enriched twice from the same third-party donor, using the overlapping peptide pools PepTivator EBV-EBNA1 and the HLA-restricted peptide pool PepTivator EBV-Select-in. The purities of the T-cell products were 47 and 46% CD3+IFN-γ+ T-cells, respectively. Two weeks after the first BV infusion, a first dosage of EBV-specific T-cells derived from this partially (8/10) HLA-matched third-party donor was administered (Supplementary Table 1).

After two courses of BV and EBV-directed T-cells, shrinkage of the lung tumors was observed in CT. Interpreting the agonizing cough and persistent dyspnea to be primarily caused by a left pulmonary lymphoma mass (Figure 1C), an individualized decision for surgery was made, and the patient underwent thoracotomy with left lower lobectomy, resulting in symptom relief. One month after chest surgery, the patient complained of increasing headaches and dizziness. Demonstration of a single enhancing lesion on brain MRI (Supplementary Figure 2) together with PCR-detection of EBV-DNA in the CSF was highly suggestive of a cerebral PTLD manifestation (biopsy refused by patient). Radiotherapy (40 Gy) was applied to the cerebral lesion.

In summary, between October 2016 and November 2017, the patient was treated with 12 courses of BV, six courses of EBV-specific T-lymphocytes, and monthly infusions of polyclonal immunoglobulins. The performance status improved continuously, and symptoms of pulmonary as well as CNS manifestations resolved completely 5 months after chest surgery and radiotherapy. Except for mild sensory polyneuropathy, the patient developed no side effects. One year following the initial PTLD diagnosis, staging by PET-CT and cMRI demonstrated CR of all disease manifestations (Figure 1D, Supplementary Figure 1B). Nearly 4 years after the initial diagnosis of AML and 3.5 years after the occurrence of PTLD, the patient remains free of hematologic disease.

## Discussion

Following alloHSCT for hematologic disease, up to 11% of patients develop PTLD, with histopathology demonstrating DLBCL in ~50% of the cases (2, 3, 11). In accordance with established risk factors (3, 11), our patient was older than 40 years and had undergone MUD transplantation in serological EBV-mismatch. Further, the patient's history of CIBD including azathioprine treatment added to the risk of PTLD by augmented immunosuppression. The risk of PTLD in this situation was significantly enhanced by *in vivo* T-cell depletion by ATG treatment. Although significant EBV DNAemia was detected at days +88 and +111, respectively, we went without preemptive rituximab, taking into consideration the spontaneous EBV-DNA decline to <5 EBV copies/ml, cessation of immunosuppressives, initial absence of clinical correlates, and increased risk of recurrent opportunistic infections.

In our patient, the rapid onset and deterioration of PTLD symptoms and the non-responsiveness to rituximab, which has been reported in 30-50% of post-alloHSCT PTLD (2, 4, 11), characterized the highly aggressive PTLD course. Even polychemotherapy was ineffective, and, only after administration of the CD30-directed immunotoxin BV disease control could be achieved. Of note, significant CD30 co-expression is observed in up to 85% of PTLD subtypes (5), making CD30 an attractive target in PTLD (1). Despite initial promising results with a 70% CR rate in PTLD (6, 7), advanced clinical trials of BV in this particular situation have not yet been reported. More importantly, the long-term efficacy of monotherapy with BV in CD30+ DLBCL arising from PTLD is undetermined.

EBV-associated PTLD is the result of impaired anti-viral T-cell activity following alloHSCT. EBV-specific cytotoxic lymphocytes (CTL) are capable of inducing strong EBV-specific cellular immune response. In the past, *in vitro*-expanded EBV-specific CTL have been infused as part of different therapeutic strategies, using both autologous and allogeneic CTL (10, 12, 13). In addition, new approaches have been developed, including the adoptive transfer of third-party virus-specific T-lymphocytes (9, 13). This approach enables T-cell generation by stimulation and selection with overlapping viral peptides (10) without the time-consuming procedure of *in vitro*-culture of CTL. Furthermore, EBV-specific T-lymphocytes can be collected from third-party donors in the situation of EBV-negative stem cell donors, as outlined in this case report. Here, it was possible to identify an adequately HLA-matched third-party donor from the alloCELL registry within 24 h and to verify donor eligibility within 3 days. Production of EBV-specific T-cells could be initiated within 2 weeks, and the patient received a total of six infusions from two production runs over a period of 8 months.

In conclusion, we report the first case of long-term control (cure) of highly aggressive EBV-PTLD including cerebral disease by combined brentuximab vedotin (BV) and adoptive EBV-specific T-cell therapy. We postulate that both rapid disease control by BV and also restoration of EBV-specific T-cell immunity were crucial components of our approach. Indeed, EBV-specific T-lymphocytes could be detected in the patient's peripheral blood one year after the last application of third-party T-cells. T cells were directed against the EBV-derived antigens used in the manufacturing process (EBNA-1, EBV-select) as well as unrelated antigens (LMP-2a), suggesting epitope spreading as part of an endogenous immune response. Considering 2-year overall survival rates of <50% (4, 11), rituximab-refractory PTLD poses a significant target for future clinical research. Various approaches, such as adoptive immunotherapy with virus-specific or chimeric antigen receptor (CAR) T-cells and also novel agents including brentuximab, have been suggested (1). However, today, there is no consensus on how to treat rituximab-refractory PTLD, especially in highly aggressive disease. In our opinion, the favorable treatment outcome in the challenging situation of our patient warrants further studies of combined BV and third-party EBV-specific T-cells in CD30+ EBV-associated PTLD.

## Data Availability Statement

All datasets generated for this study are included in the article/Supplementary Material.

## Ethics Statement

Written informed consent was obtained from the participant for the publication of this case report.

## Author Contributions

TM, CA, US, IT, ST-Z, and RS collected the data and prepared the figures and tables. TM, KS, SL, BE-V, BM-K, and RS wrote the manuscript.

### Conflict of Interest

The authors declare that the research was conducted in the absence of any commercial or financial relationships that could be construed as a potential conflict of interest.

## References

[1] Al Hamed R Bazarbachi AH Mohty M. Epstein-Barr virus-related post-transplant lymphoproliferative disease (EBV-PTLD) in the setting of allogeneic stem cell transplantation: a comprehensive review from pathogenesis to forthcoming treatment modalities. Bone Marrow Transplant. (2019). [Epub ahead of print]. 10.1038/s41409-019-0548-7. 31089285

[2] Dierickx D Habermann TM. Post-transplantation lymphoproliferative disorders in adults. N Engl J Med. (2018) 378:549–62. 10.1056/NEJMra1702693 29414277

[3] Fox CP Burns D Parker AN Peggs KS Harvey CM Natarajan S. EBV-associated post-transplant lymphoproliferative disorder following *in vivo* T-cell-depleted allogeneic transplantation: clinical features, viral load correlates and prognostic factors in the rituximab era. Bone Marrow Transplant. (2014) 49:280–6. 10.1038/bmt.2013.170 24212561

[4] DeStefano CB Desai SH Shenoy AG Catlett JP. Management of post-transplant lymphoproliferative disorders. Br J Haematol. (2018) 182:330–43. 10.1111/bjh.15263 29741774

[5] Vase MO Maksten EF Bendix K Hamilton-Dutoit S Andersen C Moller MB. Occurrence and prognostic relevance of CD30 expression in post-transplant lymphoproliferative disorders. Leukemia Lymphoma. (2015) 56:1677–85. 10.3109/10428194.2014.966242 25248878

[6] Gandhi M Ma S Smith SM Nabhan C Evens AM Winter JN Brentuximab vedotin (BV) plus rituximab (R) as frontline therapy for patients (Pts) with epstein barr virus (EBV)+ and/or CD30+ lymphoma: phase i results of an ongoing phase I-II study. Blood. (2014) 124:3096 10.1182/blood.V124.21.3096.3096

[7] Jacobsen ED Sharman JP Oki Y Advani RH Winter JN Bello CM. Brentuximab vedotin demonstrates objective responses in a phase 2 study of relapsed/refractory DLBCL with variable CD30 expression. Blood. (2015) 125:1394–402. 10.1182/blood-2014-09-598763 25573987

[8] Berger GK McBride A Lawson S Royball K Yun S Gee K. Brentuximab vedotin for treatment of non-Hodgkin lymphomas: A systematic review. Crit Rev Oncol Hematol. (2017) 109:42–50. 10.1016/j.critrevonc.2016.11.009 28010897 PMC5218629

[9] Schultze-Florey RE Tischer S Kuhlmann L Hundsdoerfer P Koch A Anagnostopoulos I. Dissecting epstein-barr virus-specific T-cell responses after allogeneic EBV-specific T-cell transfer for central nervous system posttransplant lymphoproliferative disease. Front Immunol. (2018) 9:1475. 10.3389/fimmu.2018.01475 29997626 PMC6030255

[10] Eiz-Vesper B Maecker-Kolhoff B Blasczyk R. Adoptive T-cell immunotherapy from third-party donors: characterization of donors and set up of a T-cell donor registry. Front Immunol. (2012) 3:410. 10.3389/fimmu.2012.00410 23372567 PMC3556568

[11] Garcia-Cadenas I Yanez L Jarque I Martino R Perez-Simon JA Valcarcel D. Frequency, characteristics, and outcome of PTLD after allo-SCT: A multicenter study from the Spanish group of blood and marrow transplantation (GETH). Eur J Haematol. (2019) 102:465–71. 10.1111/ejh.13226 30828868

[12] Icheva V Kayser S Wolff D Tuve S Kyzirakos C Bethge W. Adoptive transfer of epstein-barr virus (EBV) nuclear antigen 1-specific t cells as treatment for EBV reactivation and lymphoproliferative disorders after allogeneic stem-cell transplantation. J Clin Oncol. (2013) 31:39–48. 10.1200/JCO.2011.39.8495 23169501

[13] Haque T Wilkie GM Jones MM Higgins CD Urquhart G Wingate P. Allogeneic cytotoxic T-cell therapy for EBV-positive posttransplantation lymphoproliferative disease: results of a phase 2 multicenter clinical trial. Blood. (2007) 110:1123–31. 10.1182/blood-2006-12-063008 17468341

