# Supplementary material for: Refractory Epstein-Barr Virus (EBV)-Related Post-transplant Lymphoproliferative Disease: Cure by Combined Brentuximab Vedotin and Allogeneic EBV-Specific T-Lymphocytes
Source: Front Med (Lausanne). 2019 Dec 18;6:295. doi: 10.3389/fmed.2019.00295 (PMC6930172; doi:10.3389/fmed.2019.00295)
Supplement: Supplementary file 1 [file Data_Sheet_1.pdf]

SUPPLEMENTARY FIGURES

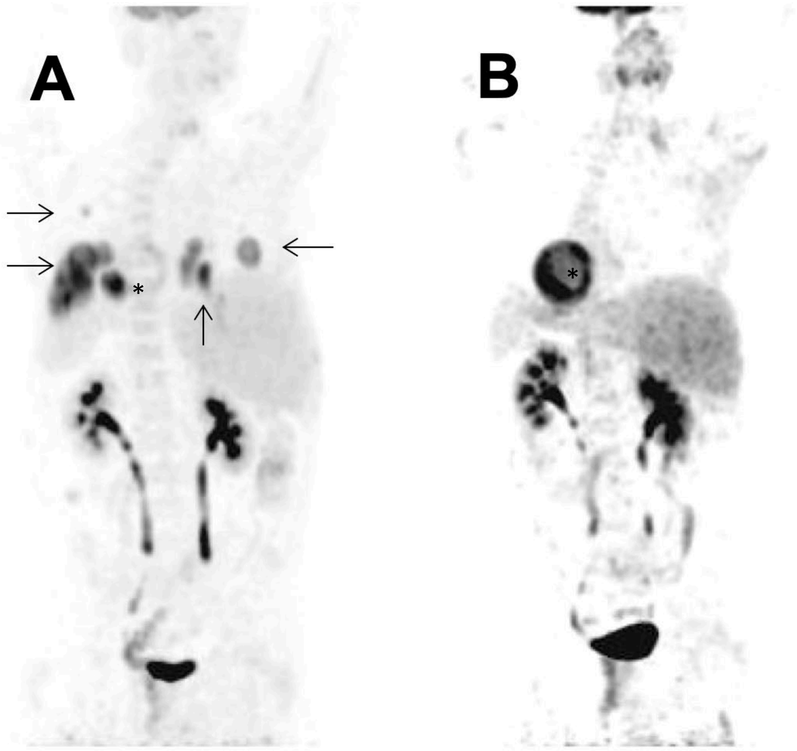

Supplementary Figure 1

PET-CT during disease course. **A:** Multiple PTLD manifestations at day +228 post alloHSCT, **B:** Complete remission of PTLD one year after diagnosis; *****: FDG-activity heart muscle.

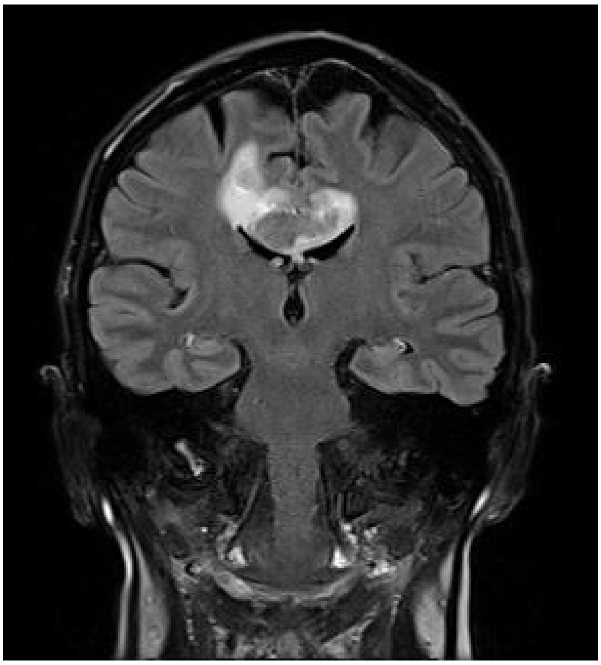

Supplementary Figure 2

Cerebral MRI at day +300 post alloHSCT. Lymphoma typical lesion interpreted as PTLD manifestation (T1, gadolinium)

Supplementary Tables

	HLA-A		HLA-B		HLA-C		HLA-DRB1		HLA-DQ	
**Patient**	01:01P	03:01P	08:01P	35:01P	07:01P	04:01P	*01:01P*	03:01P	*05:01P*	02:01P
**T-cell Donor**	01:01:01G	03:01:01G	08:01:01G	35:01:01G	07:01P	04:01:01G	*15:01P*	03:01P	*06:02P*	02:01P

Supplementary Table 1A

Matching of HLA-alleles in PTLD patient following alloHSCT and third-party donor of EBV-specific T-lymphocytes.

time point of administration	*EBNA1/Select-specific T-cell dosage*
November 2016	1.0 x 10^4 CD3+ cells/kg *
December 2016	0.5 x 10^4 CD3+ cells/kg $^+$
January 2017	0.5 x 10^4 CD3+ cells/kg $^+$
March 2017	1.0 x 10^4 CD3+ cells/kg *
May 2017	0.5 x 10^4 CD3+ cells/kg $^+$
July 2017	0.5 x 10^4 CD3+ cells/kg $^+$

* *fresh,* $^+$ *cryopreserved*

Supplementary Table 1B

Scheduling and dosing of EBV-specific T-cell administration. EBV-specific T-cells were selected twice from the same unrelated donor and were prepared based on interferon-γ release following stimulation with EBV EBNA1 and EBV Select peptide pools, as previously reported[9,10].
